# Trainee-Physician Milestones Ratings and Patient Experience Surveys in Early Unsupervised Practice

**DOI:** 10.1001/jamanetworkopen.2025.36380

**Published:** 2025-10-08

**Authors:** Jenny X. Chen, Deirdre Mylod, Yuezhou Jing, Madeleine Kerschner, Bruce Trock, Steve Meth, Kenji Yamazaki, Sean O. Hogan, Misop Han

**Affiliations:** 1 Department of Otolaryngology-Head and Neck Surgery, Johns Hopkins School of Medicine, Baltimore, Maryland; 2 Press Ganey Associates, Inc., South Bend, Indiana; 3 James Buchanan Brady Urological Institute and Department of Urology, Johns Hopkins School of Medicine, Baltimore, Maryland; 4 Johns Hopkins Medicine, Baltimore, Maryland; 5 Accreditation Council for Graduate Medical Education, Chicago, Illinois

## Abstract

**Importance:**

Milestones ratings have been used to assess resident physicians for more than a decade, but little is known as to whether there are posttraining implications of ratings for patient experiences.

**Objective:**

To investigate the association of residents’ Milestones ratings of professionalism and interpersonal and communication skills (ICS) with patient experience survey results in the first year of unsupervised practice.

**Design, Setting, and Participants:**

This retrospective cohort study included physicians who completed residency training at Accreditation Council for Graduate Medical Education–accredited programs between July 1, 2015, and June 30, 2019, and subsequently worked at facilities that collected Clinician & Group-Consumer Assessments of Healthcare Providers and Systems Surveys (CG-CAHPS). Milestones ratings from adult primary care training programs (internal medicine, family medicine, and obstetrics and gynecology) and patient survey data were linked for physicians who had more than 30 CG-CAHPS results in their first year of unsupervised practice. Physician characteristics (sex and specialty), CG-CAHPS patient respondent demographics (sex, race, and language spoken at home), and a CG-CAHPS question about a facility-related experience were collected for multivariable linear regression analyses. Data were analyzed from February 1, 2024 to April 10, 2025.

**Exposures:**

Mean professionalism and ICS scores in Milestones evaluations collected 6 months before the end of training. Physicians were categorized into those with higher (≥3.5) or lower (<3.5) mean scores.

**Main Outcomes and Measures:**

CG-CAHPS Top Box scores for 6 questions pertaining to physician behaviors and the overall clinician rating. (Top Box scores denote the percentage of patients who selected the most favorable response option.)

**Results:**

A total of 1349 physicians (753 [55.8%] aged 26-30 years; 804 [59.6%] female) were included in the analysis. Higher mean professionalism or ICS Milestones ratings were associated with higher Top Box scores across all CG-CAHPS questions pertaining to physician-related experiences (eg, adjusted mean difference in physician’s knowledge of the patient’s medical history: professionalism, 2.9 [95% CI, 1.4-4.5] percentage points; *P* < .001; ICS, 3.2 [95% CI, 1.5-4.9] percentage points; *P* < .001) as well as overall physician ratings (adjusted mean difference: professionalism, 2.9 [95% CI, 1.4-4.3] percentage points; *P* < .001; ICS, 3.5 [95% CI, 2-5.1] percentage points; *P* < .001) in multivariable linear regression models.

**Conclusions and Relevance:**

In this cohort study of physicians who completed residency in adult primary care specialties, those with higher mean professionalism and ICS Milestones ratings in training received better patient experience survey ratings in their first year of unsupervised practice. These findings suggest that trainees with lower ratings may benefit from intervention.

## Key Points

**Question:**

Are professionalism and interpersonal and communication skills Milestones ratings from the final 6 months of training associated with patient experience survey results for physicians in adult primary care specialties during early unsupervised practice?

**Findings:**

In this cohort study of 1349 physicians, higher professionalism and interpersonal and communication skills Milestones ratings were significantly associated with better patient survey ratings of physician behavior and communication during the first year of practice.

**Meaning:**

These findings suggest that individuals with lower Milestones ratings may benefit from intervention during training or early in their careers.

## Teaser

This cohort study examines the associations of residents’ Milestones ratings of professionalism and interpersonal and communication skills during the final months of training with patient experience survey results during the first year of unsupervised practice.

## Introduction

Since 2013, training programs accredited by the Accreditation Council for Graduate Medical Education (ACGME) have evaluated resident physicians using Milestones ratings.^1,2^ Although Milestones are specialty-specific, they all map to the 6 core competencies: medical knowledge, patient care, practice-based learning and improvement, systems-based practice, interpersonal and communication skills (ICS), and professionalism. Despite their widespread use across specialties and programs in the US, there remains a paucity of research examining the relationship between Milestones ratings and posttraining outcomes, including measures of patient experience.

A 2023 study by Han et al^3^ found that Milestones ratings in professionalism and ICS were associated with unsolicited patient complaints during early independent practice, as recorded in the national Patient Advocacy Reporting System. However, a lack of complaints does not necessarily indicate positive patient experiences. In this study, we examined associations between trainees’ Milestones ratings and the experiences of their patients in early unsupervised practice, as measured with the Consumer Assessment of Healthcare Providers and Systems Clinician and Group Survey (CG-CAHPS). The CG-CAHPS asks patients to rate various aspects of care: (1) timeliness of appointments, care, and information; (2) physician behaviors; (3) experiences with office staff; and (4) patients’ overall ratings of their physician.^4^ These surveys are validated, reliable tools that are widely used by organizations and health care consumers to assess clinicians and practices.^5^

This study investigated the association of the professionalism and ICS Milestones ratings of trainees in adult primary care specialties with the patient survey responses they received during their first year of practice. Professionalism and communication are thought to be essential components of effective medical practices, directly influencing patient safety and quality. We hypothesized that there is an association between in-training assessments 6 months prior to graduation and performance in early unsupervised practice.

## Methods

### Study Design

This cohort study was deemed exempt from review and informed consent by the institutional review boards at Johns Hopkins University per 45 CFR 46.104(d)(2)(ii). The study is reported following the Strengthening the Reporting of Observational Studies in Epidemiology (STROBE) reporting guideline. We performed a retrospective study of trainees in ACGME-accredited adult primary care training programs (internal medicine [IM], family medicine [FM], and obstetrics and gynecology [OB-GYN]) who finished their training between July 1, 2015, and June 30, 2019.

### Data Sources

Milestones ratings are reported semiannually by ACGME-accredited graduate medical education programs. The ACGME collects Milestones ratings as well as trainee identifiers (eg, sex, year of training completion) and training program attributes. These data were linked to CG-CAHPS responses collected by Press Ganey. Press Ganey supports more than 3700 sites with 507 000 clinicians nationally, collecting nearly 8 million survey responses annually from patients evaluating their care experiences. The ACGME provided a dataset with Milestones ratings to Press Ganey researchers, who linked them to clinician-level aggregations of patient experience data using National Provider Identifier numbers. Following linkage, identifying and demographic data were removed prior to data analysis to ensure that no unique combination could identify individuals.

### Study Participants

We included all trainees who completed adult primary care residencies during the study period. If a trainee completed more than 1 program, we examined the last training program completed. We then narrowed the cohort to trainees who went on to practice at institutions that collected Press Ganey surveys and collected at least 30 CG-CAHPS surveys in their first year of practice. This criterion, recommended by Press Ganey researchers, is based on the Central Limit Theorem, which states that sample sizes of 30 or larger have sufficiently stable, normally distributed outcomes.^6^ A diagram of the steps of cohort development is provided in the Figure. Of 51 147 trainees from the study period with Milestones ratings available, 46 669 were excluded because they worked at sites after graduation that did not collect Press Ganey surveys. An additional 3129 trainees were excluded for having fewer than 30 patient survey responses in their first year of practice.

**Figure.**
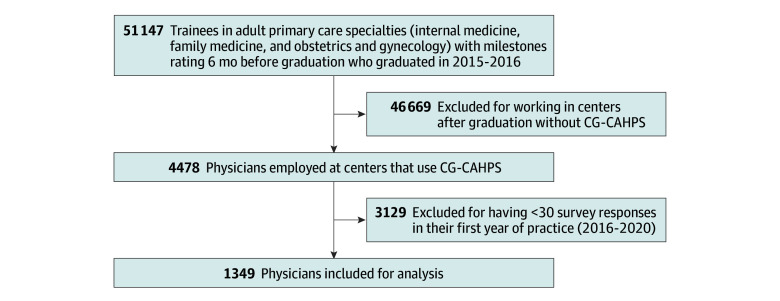
Physician Cohort Development CG-CAHPS indicates Clinician & Group-Consumer Assessments of Healthcare Providers and Systems Surveys.

### ICS and Professionalism Milestones Ratings

During the study period, the Milestones ratings had minor variations across specialties.^7^ FM and OB-GYN trainees were rated from 1 to 5, where 1 indicated novice performance and 5, expert or aspirational performance.^8^ IM Milestones were rated from 0 to 4 ,where 0 indicated critical deficiencies, 1 and 2 had no labels, 3 indicated ready for unsupervised practice, and 4, aspirational. As such, IM ICS and professionalism Milestones were cross walked to FM and OB-GYN Milestones and rescaled by adding a point to each of the ratings, such that 4 represented the graduation target and 5 represented aspirational performance for all 3 specialties. Each scale had a half-point option (eg, 2.5). Full descriptions of Milestones (version 1.0) for FM, IM, and OB-GYN have been published elsewhere.^9^

The exposure for this cohort study was the mean professionalism or ICS domain ratings 6 months before graduation, as this was the final point at which a program could identify low performers and intervene. Penultimate assessments also show greater statistical variation than ratings at graduation; this line of reasoning has been used in prior publications comparing Milestones with patient outcomes.^10,11^ The ACGME recommends a developmental goal of 4 or ready for unsupervised practice for trainees at the time of graduation. As we were examining ratings 6 months prior to graduation, we chose to dichotomize trainees into 2 groups: those with higher ratings (≥3.5) and those with lower ratings (<3.5), as has been described elsewhere.^10^ Not all specialties have the same number of Milestones for each competency (eg, FM has 4 professionalism Milestones whereas OB-GYN has 3), and so we used mean Milestones ratings for each competency.

### Top Box CG-CAHPS Scores

The CG-CAHPS visit experience survey is a standardized instrument administered by Press Ganey. The survey asks patients about timeliness of care, interactions with office staff, physician behaviors, and an overall rating of the physician. For this study, questions about physician behavior and communication, as well as the overall rating of the physician, were isolated as the primary outcomes, as these are most directly attributable to physicians’ interactions with patients. For each question, the physician’s Top Box score was calculated as the proportion of respondents giving the most favorable response.^12^ For example, if a physician receives a response of always from 70% of their patients for a question rated on a 4-point scale (never, sometimes, usually, always), their Top Box score would be 70%.

### Statistical Analysis

ACGME Milestones ratings and CG-CAHPS responses were linked for 1349 physicians eligible for analysis. The demographics of trainees included in the analysis sample and trainees excluded from the analysis were compared to identify potential sample selection bias. Descriptive statistics of the characteristics of 1349 trainees and their distributions within 2 tiers of Milestones ratings (<3.5 and ≥3.5) were reported and proportions were compared using χ^2^ tests. Similarly, descriptive statistics of CG-CAHPS ratings of trainees with various demographic characteristics were reported and compared by using Kruskal-Wallis tests. The association between CG-CAHPS Top Box scores and Milestones ratings were assessed using multiple linear regression models adjusted for trainee characteristics (specialty and sex), mean professionalism and ICS Milestones ratings for graduates from 2015 to 2019 at each program, summary demographics for the sample of patients evaluating clinicians (patient biological sex as listed in the medical record, self-reported race, and language spoken at home), as well as patient evaluations of a non–physician-focused CG-CAHPS question asking whether “clerks and receptionists were courteous and respectful.” For multiple linear regressions, the adjusted mean difference in CG-CAHPS Top Box ratings for a difference in Milestones ratings were reported along with their 95% CIs.

To contextualize differences in CG-CAHPS scores between groups, Top Box scores were compared to the Press Ganey CG-CAHPS national benchmarking distribution for IM during the second half of 2019 using unpublished internal data from Press Ganey. All tests were 2-sided, and statistical significance was set at *P* < .05. Analyses were completed using SAS software version 9.4 (SAS institute Inc) from February 1, 2024, to April 10, 2025.

## Results

The final study cohort included 1349 physicians (753 [55.8%] aged 26-30 years; 804 [59.6%] female). Most physicians specialized in FM (906 physicians [67.2%]) while the remainder specialized in IM (308 physicians [22.8%]) or OB-GYN (135 physicians [10.0%]). Comparing the demographic characteristics of physicians who met the inclusion criteria for this study with those of physicians excluded from the study who had Milestones ratings available (eTable 1 in Supplement 1), the distribution of physicians in different age ranges was similar. However, there were more females among the included physicians compared with excluded physicians (59.6% vs 49.2%). There were also more physicians in FM (67.2% vs 27.5%), and fewer physicians in IM (22.8% vs 62.2%) within the study cohort as compared with excluded physicians. The mean (SD) professionalism Milestones rating among physicians who met the study inclusion criteria was 3.70 (0.56) and the mean (SD) ICS Milestones rating was 3.77 (0.51), which was slightly lower than the professionalism and ICS ratings of physicians who did not meet study inclusion criteria (mean [SD], 3.79 [0.51] and 3.83 [0.47], respectively; *P* < .001) (eTable 1 in Supplement 1).

Among study participants, the proportions of those with lower (<3.5) or higher (≥3.5) Milestones ratings for professionalism scores were not significantly different across age ranges or graduation years but were significantly different for sex and specialty (Table 1). Ratings for ICS scores were different only for different specialties (Table 1).

**Table 1.** Distribution of Milestones Ratings for the Physician Cohort by Demographic and Training Characteristics

Characteristic	No. (row %) (N = 1349)					
	Mean professionalism ratings			Mean ICS ratings		
	<3.5	≥3.5	*P* value^a^	<3.5	≥3.5	*P* value^a^
Age, y						
26-30	129 (17.2)	624 (82.8)	.45	95 (12.6)	658 (87.4)	.10
31-35	95 (20.0)	380 (80.0)		81 (17.0)	394 (83.0)	
≥36	22 (18.2)	99 (81.8)		17 (14.0)	104 (86.0)	
Sex						
Female	133 (16.6)	671 (83.4)	.02	111 (13.8)	693 (86.2)	.34
Male	112 (21.4)	410 (78.6)		82 (15.8)	440 (84.2)	
Missing^b^	1 (4.4)	22 (95.6)		0	23 (100)	
Graduation year						
2015	2 (14.2)	12 (85.8)	.21	1 (7.2)	13 (92.8)	.39
2016	68 (21.0)	256 (79.0)		46 (14.2)	278 (85.8)	
2017	49 (14.2)	295 (85.8)		40 (11.6)	304 (88.4)	
2018	72 (19.6)	297 (80.4)		57 (15.4)	312 (84.6)	
2019	55 (18.4)	243 (81.6)		49 (16.4)	249 (83.6)	
Specialty						
Family medicine	213 (23.6)	693 (76.4)	<.001	161 (17.8)	745 (82.2)	<.001
Internal medicine	24 (7.8)	284 (92.2)		24 (7.8)	284 (92.2)	
Obstetrics and gynecology	9 (6.6)	126 (93.4)		8 (6.0)	127 (94.0)	

Abbreviation: ICS, interpersonal and communication skills.

^a^ χ^2^ tests for proportions.

^b^ Missing sex responses were excluded due to small cell sizes.

Overall, 87 885 patient surveys were analyzed after visits with the physician cohort. For questions related to the physician’s behavior and communication, the Top Box CG-CAHPS scores were highest for the question asking whether physicians showed respect for what the patient said (mean [SD] 95.6% [4.4 percentage points]) and lowest for the question asking whether the physician knew important information about the patient’s medical history (mean [SD] 77.6% [9.3 percentage points]). The mean (SD) overall Top Box score for physicians in the cohort was 83.1% (8.8 percentage points). Ratings of physician behaviors were significantly different across different age groups for all 6 questions. Select questions also differed across physician specialties and graduation years (Table 2).

**Table 2.** Top Box Scores for CG-GAHPS Questions Regarding Physician Behavior by Demographic and Training Characteristics

Physician characteristic	CG-CAHPS Top Box scores^a^											
	Physician explained in way you understand, mean (SD)	*P* value^b^	Physician showed respect for what you say, mean (SD)	*P* value^b^	Physician spent enough time with you, mean (SD)	*P* value^b^	Physician knew important info/ medical history, mean (SD)	*P* value^b^	Physician listened carefully to you, mean (SD)	*P* value^b^	Overall physician rating, mean (SD)	*P* value^b^
Age range, y												
26-30	94.2 (5.1)	<.001	95.6 (4.4)	.003	94.1 (5.1)	.02	77.6 (9.3)	.002	94.6 (4.9)	.002	83.1 (8.8)	<.001
31-35	93.7 (5)		95.1 (4.4)		93.6 (5.3)		76.7 (9.6)		94.2 (4.9)		82.3 (9.2)	
>35	92.0 (6.2)		93.7 (5.8)		92.5 (6)		73.9 (10.6)		92.5 (6.5)		79.0 (10.4)	
Sex												
Female	94.0 (5.1)	.28	95.4 (4.5)	.63	93.6 (5.5)	.69	76.9 (9.6)	.85	94.3 (5.1)	.85	82.8 (8.9)	.33
Male	93.5 (5.4)		95.0 (4.8)		93.9 (5)		76.9 (9.7)		94.2 (5)		81.9 (9.5)	
Missing	93.8 (5.8)		95.0 (4.1)		93.5 (5.3)		77.5 (9.6)		93.8 (5.5)		82.0 (9.4)	
Graduation year												
2015	94.5 (3.6)	.38	96.8 (3.1)	.19	93.1 (4.3)	.48	75.8 (7.5)	.001	95.5 (3.2)	.29	81.0 (5.2)	<.001
2016	93.5 (5.4)		95.0 (4.6)		93.4 (5.4)		75.6 (9.8)		93.9 (5.4)		80.8 (9.8)	
2017	93.5 (5.6)		95.0 (4.8)		93.5 (5.6)		76.2 (9.4)		93.9 (5.4)		82.1 (8.9)	
2018	94.2 (5.1)		95.5 (4.5)		93.9 (5.2)		78.1 (9.7)		94.5 (4.9)		82.6 (9.5)	
2019	93.9 (4.8)		95.4 (4.5)		94.1 (4.9)		78.0 (9.2)		94.7 (4.7)		84.3 (8.1)	
Specialty												
Family medicine	93.9 (5)	.005	95.3 (4.5)	.26	93.8 (5.2)	.37	76.6 (9.6)	.20	94.4 (5)	.19	82.6 (9.1)	.01
Internal medicine	93.0 (5.7)		94.9 (4.8)		93.5 (5.2)		77.3 (9.5)		93.8 (5.3)		81.2 (9.5)	
Obstetrics and gynecology	94.6 (5.2)		95.5 (4.8)		93.9 (5.9)		77.9 (9.3)		94.5 (5.3)		83.7 (8.9)	

^a^ Mean scores from 87 885 patient surveys.

^b^ *P* values from Kruskal-Wallis Tests.

In multivariable linear regression models, higher professionalism and ICS ratings (≥3.5 vs <3.5) were associated with better Top Box scores for all 6 CG-CAHPS questions about physician behavior and communication (Table 3 and Table 4). Full models are available in eTable 2 and eTable 3 in Supplement 1. Compared with physicians with lower professionalism ratings, those with higher score had CG-CAHPS Top Box scores that were 1.4 to 2.9 points higher (Table 3). Similarly, compared with physicians with lower ICS ratings, those with higher ratings had CG-CAHPS Top Box scores that were 1.5 to 3.5 points higher (Table 4). For both professionalism and ICS ratings, differences in Top Box scores were highest for CG-CAHPS questions about the physician’s knowledge of the patient’s medical history (adjusted mean difference: professionalism, 2.9 [95% CI, 1.4-4.5] percentage points; *P* < .001; ICS, 3.2 [95% CI, 1.5-4.9] percentage points; *P* < .001) and the overall physician rating (adjusted mean difference: professionalism, 2.9 [95% CI, 1.4-4.3] percentage points; *P* < .001; ICS, 3.5 [95% CI, 2-5.1] percentage points; *P* < .001). In multivariable analyses, patient characteristics, such as the proportion of female, non-White, and non–English speaking patients, were also associated with Top Box scores for select CG-CAHPS questions (eTable 2 and eTable 3 in Supplement 1).

**Table 3.** Associations of Professionalism Milestones Ratings With Top Box Scores for CG-CAPHS Questions Regarding Physician Behavior

Mean professionalism score	CG-CAPHS Top Box score, adjusted mean (95% CI), %^a^	Adjusted mean difference (95% CI), %	*P* value
Physician explained in way you understand			
<3.5	92.7 (91.6-93.7)	0 [Reference]	<.001
≥3.5	94.3 (93.5-95.1)	1.6 (0.8-2.4)	
Physician showed respect for what you say			
<3.5	94.1 (93.2-95)	0 [Reference]	<.001
≥3.5	95.6 (94.9-96.2)	1.5 (0.7-2.2)	
Physician spent enough time with you			
<3.5	92.7 (91.6-93.7)	0 [Reference]	.001
≥3.5	94 (93.3-94.8)	1.4 (0.6-2.2)	
Physician knew important information/medical history			
<3.5	74.9 (73-76.9)	0 [Reference]	<.001
≥3.5	77.9 (76.4-79.4)	2.9 (1.4-4.5)	
Physician listened carefully to you			
<3.5	93.1 (92.1-94.1)	0 [Reference]	<.001
≥3.5	94.5 (93.8-95.3)	1.4 (0.6-2.2)	
Overall physician rating			
<3.5	80.4 (78.5-82.3)	0 [Reference]	<.001
≥3.5	83.3 (81.9-84.7)	2.9 (1.4-4.3)	

Abbreviation: CG-CAHPS, Clinician & Group-Consumer Assessments of Healthcare Providers and Systems Surveys.

^a^ Adjusted for physician gender, specialties, mean residency program Milestones ratings, as well as patient gender, race, language spoken at home, and their rating on a facility-centered question asking whether “clerks and receptionists were courteous and respectful.”

**Table 4.** Associations of Interpersonal and Communication Skills Milestones Ratings With Top Box Scores for CG-CAPHS Questions Regarding Physician Behavior

Mean ICS rating	CG-CAPHS Top Box score, adjusted mean (95% CI), %^a^	Adjusted mean difference (95% CI), %	*P* value
Physician explained in way you understand			
<3.5	92.1 (91-93.3)	0 [Reference]	<.001
≥3.5	94.2 (93.5-95)	2.1 (1.2-3)	
Physician showed respect for what you say			
<3.5	93.7 (92.7-94.7)	0 [Reference]	<.001
≥3.5	95.5 (94.8-96.2)	1.8 (1-2.6)	
Physician spent enough time with you			
<3.5	92.5 (91.3-93.6)	0 [Reference]	<.001
≥3.5	94 (93.2-94.8)	1.5 (0.6-2.4)	
Physician knew important info/medical history			
<3.5	74.6 (72.4-76.7)	0 [Reference]	<.001
≥3.5	77.8 (76.3-79.3)	3.2 (1.5-4.9)	
Physician listened carefully to you			
<3.5	92.8 (91.7-93.9)	0 [Reference]	<.001
≥3.5	94.5 (93.7-95.3)	1.7 (0.8-2.6)	
Overall physician rating			
<3.5	79.7 (77.6-81.7)	0 [Reference]	<.001
≥3.5	83.2 (81.8-84.6)	3.5 (2-5.1)	

Abbreviations: CG-CAHPS, Clinician & Group-Consumer Assessments of Healthcare Providers and Systems Surveys; ICS, interpersonal and communication skills.

^a^ Adjusted for trainee gender, specialties, mean residency program Milestones ratings, as well as patient gender, race, language spoken at home, and their rating on a facility-centered CG-CAHPS question asking whether “clerks and receptionists were courteous and respectful.”

To contextualize the magnitude of these differences, Top Box scores in this cohort were compared with unpublished Press Ganey CG-CAHPS national benchmarking distribution for IM in 2019. For the 4 questions related to the interpersonal behaviors of the physician (ie, explained in a way you understand, showed respect, spent enough time, and listened carefully), the mean difference in percentile ranks for the Top Box scores of physicians with higher vs lower Milestones ratings was 14 percentile ranks for professionalism and 16 percentile ranks for ICS. For the question asking for an overall physician rating, the mean difference in percentile ranks for Top Box scores was 6 percentile ranks for professionalism and 7 percentile ranks for ICS. The question regarding knowing important medical history had the smallest spread in Top Box scores, and the difference was only 1 percentile rank for both professionalism and ICS for those with higher or lower Milestones ratings.

Sensitivity analyses were conducted using adjusted mean CG-CAHPS scores instead of Top Box scores and the same associations with Milestones ratings of professionalism and ICS were found for all 6 CG-CAHPS questions (eTable 4 and eTable 5 in Supplement 1). Sensitivity analyses conducted with a Milestones rating threshold of 4 also found an association of professionalism and ICS ratings with all 6 CG-CAHPS questions (eTable 6 and eTable 7 in Supplement 1).

## Discussion

In this cohort study, we linked the ACGME Milestones ratings in the professionalism and ICS competencies of senior trainees in adult primary care specialties to the results of CG-CAHPS patient experience surveys collected in their first year of unsupervised practice. We found that there were significant associations of professionalism and ICS Milestones ratings with patient responses to all questions about physician behavior and communication, as well as overall physician rating.

This study adds to the evolving literature that connects competency-based evaluations collected in training to outcomes of consequence in early years of practice. Han et al^3^ also detected an association of low professionalism and ICS ratings with unsolicited patient complaints. A single-institution study of general surgery residents found there was an association between ICS scores and select CG-CAHPS questions for trainees even during residency.^13^ We have now confirmed this association in a larger study following trainees into their early practice, once again highlighting the importance of programmatic assessment within graduate medical education.

While there may be an association between Milestones ratings and patient-reported experiences, the association between Milestones ratings and patient clinical outcomes remains less clear. In general surgery, Kendrick et al^10^ found no association of composites of Milestones ratings 6 months before graduation with complications or death within 30 days of operations. Select composites of Milestones ratings designed to describe operative performance, professionalism, and leadership were also not associated with patient outcomes. In contrast, Wirtalla et al^14^ used machine learning to examine a larger collection of ways in which patterns of Milestones data from the final 4 biannual assessments of trainees before graduation might be associated with early patient outcomes for general surgeons.^14^ They found that individual Milestones under the competencies of patient care, professionalism, and systems-based practice during postgraduate year 4 were associated with risk-adjusted death and patient morbidity. Smith et al^11^ also found that a composite score of Milestones ratings across competencies collected 6 months prior to graduation was associated with complications after endovascular aortic aneurysm repair for recent graduates of vascular surgery programs.

### Implications for Graduate Medical Education

Milestones represent 2 of the 5 core components of competency-based medical education^15^ in that they are clearly articulated competencies that are sequenced progressively. As such, Milestones must be combined with tailored learning experiences, competency-focused instruction, and programmatic assessment to optimize their impact on learning. For example, residency programs should set achievement benchmarks throughout training and tailor individualized learning plans for residents who need further intervention. There is evidence that mentorship, coaching, and even simulation may be effective for teaching and remediating professionalism and ICS.^16,17^

### Limitations

This study has several limitations. First, we treated Milestones ratings under each competency as equivalent across multiple specialties, despite variations in wording and the numbers of distinct Milestones used. We also did not adjust for differences in Milestones ratings over time even though we included early years of implementation, as prior studies have indicated that there is limited drift in ratings.^18^ We reasoned that the means of Milestones ratings would allow us to make observations about the broader competency, which maintains a consistent definition across specialties. Analyzing specialties separately would have further limited our sample size. Second, our focus was on professionalism and ICS because of their clear relevance to CG-CAHPS questions, but other domains may also influence patient experiences. Future studies may even map specific subcompetencies to patient experiences. Third, we only included physicians employed at organizations that use Press Ganey to collect CG-CAHPS data. However, it may be that physicians working at institutions that do not use CG-CAHPS fundamentally differ from those included in the study. Fourth, our analyses were limited to physicians with at least 30 CG-CAHPS responses in their first year of practice, which decreased the size of the cohort included in the study and increases the risk for selection bias but improved the reliability of the outcome data collected. Data for physicians with fewer ratings were not made available. Consequently, we focused on adult primary care specialties, as these physicians were the best represented among those who met inclusion criteria. Moreover, FM physicians represented more than half the cohort, which may also introduce bias; for example, FM physicians may have more longitudinal relationships with patients than other outpatient physicians, which may impact patient experiences. Additional research is needed to determine whether these findings are generalizable. Fifth, not all factors that impact patient experiences could be analyzed. We could not account for variation across variables such as precise physician ages, patient or physician race and ethnicity, training program characteristics, or the regional impact of COVID-19, as we did not have this information. Select data were exchanged under a data use agreement that prioritized the anonymity of studied physicians. As such, proxy variables were substituted to account for variation across medical institutions (eg, program mean of Milestones domain ratings) or patient populations (eg, language spoken at home). There is evidence that a variety of factors, such as concordance in physician-patient sex, race, ethnicity, or language, as well as various institutional characteristics, affect patient experiences.^19,20,21^ Furthermore, Milestones ratings from other time points in training could have been chosen, and using penultimate ratings may not capture remediation efforts in the final 6 months. However, penultimate ratings have proximity to unsupervised practice while minimizing the leniency bias associated with ratings at graduation.^10,14^ For example, program directors might misconstrue level 4 Milestones ratings as a graduation requirement rather than a recommended goal.

## Conclusions

In this retrospective cohort study of recent graduates of adult primary care residency programs, higher professionalism and ICS Milestones ratings during training were associated with better scores on patient experience surveys of these physician’s behaviors and communication in early unsupervised practice. Poor Milestones ratings in these domains during training may justify tailored interventions.

## Notes

Supplement 1. **eTable 1.** Comparing the demographics of included and excluded physician cohorts who completed training at ACGME accredited programs between July 1, 2015, and June 30, 2019 **eTable 2.** Multivariable linear regression models correlating Top Box scores for CG-CAHPS questions regarding patient behavior with ACGME Milestones professionalism domain ratings 6 months prior to graduation **eTable 3.** Multivariable linear regression models correlating Top Box scores for CG-CAHPS questions regarding patient behavior with ACGME Milestones interpersonal and communication skills domain ratings 6 months prior to graduation **eTable 4.** Multivariable linear regression models correlating adjusted mean scores for CG-CAHPS questions regarding patient behavior with ACGME Milestones professionalism domain ratings 6 months prior to graduation **eTable 5. **Multivariable linear regression models correlating adjusted mean scores for CG-CAHPS questions regarding patient behavior with ACGME Milestones interpersonal and communication skills domain ratings 6 months prior to graduation **eTable 6.** Multivariable linear regression models correlating Top Box scores for CG-CAHPS questions regarding patient behavior with ACGME Milestones professionalism domain ratings 6 months prior to graduation **eTable 7.** Multivariable linear regression models correlating Top Box scores for CG-CAHPS questions regarding patient behavior with ACGME Milestones interpersonal and communication skills domain ratings 6 months prior to graduation

Supplement 2.
Data Sharing Statement
