# Supplementary material for: Trainee-Physician Milestones Ratings and Patient Experience Surveys in Early Unsupervised Practice
Source: JAMA Netw Open. 2025 Oct 8;8(10):e2536380. doi: 10.1001/jamanetworkopen.2025.36380 (PMC12509016; doi:10.1001/jamanetworkopen.2025.36380)
Supplement: Supplement 1. — eTable 1. Comparing the demographics of included and excluded physician cohorts who completed training at ACGME accredited programs between July 1, 2015, and June 30, 2019 eTable 2. Multivariable linear regression models correlating Top Box scores for CG-CAHPS questions regarding patient behavior with ACGME Milestones professionalism domain ratings 6 months prior to graduation eTable 3. Multivariable linear regression models correlating Top Box scores for CG-CAHPS questions regarding patient behavior with ACGME Milestones interpersonal and communication skills domain ratings 6 months prior to graduation eTable 4. Multivariable linear regression models correlating adjusted mean scores for CG-CAHPS questions regarding patient behavior with ACGME Milestones professionalism domain ratings 6 months prior to graduation eTable 5. Multivariable linear regression models correlating adjusted mean scores for CG-CAHPS questions regarding patient behavior with ACGME Milestones interpersonal and communication skills domain ratings 6 months prior to graduation eTable 6. Multivariable linear regression models correlating Top Box scores for CG-CAHPS questions regarding patient behavior with ACGME Milestones professionalism domain ratings 6 months prior to graduation eTable 7. Multivariable linear regression models correlating Top Box scores for CG-CAHPS questions regarding patient behavior with ACGME Milestones interpersonal and communication skills domain ratings 6 months prior to graduation [file jamanetwopen-e2536380-s001.pdf]

Supplemental Online Content

Chen JX, Mylod D, Jing Y, et al. Trainee-physician Milestones ratings and patient experience surveys in early unsupervised practice. *JAMA Netw Open*. 2025;8(10):e2536380.
doi:10.1001/jamanetworkopen.2025.36380

eTable 1. Comparing the demographics of included and excluded physician cohorts who completed training at ACGME accredited programs between July 1, 2015, and June 30, 2019

eTable 2. Multivariable linear regression models correlating Top Box scores for CG-CAHPS questions regarding patient behavior with ACGME Milestones professionalism domain ratings 6 months prior to graduation

eTable 3. Multivariable linear regression models correlating Top Box scores for CG-CAHPS questions regarding patient behavior with ACGME Milestones interpersonal and communication skills domain ratings 6 months prior to graduation

eTable 4. Multivariable linear regression models correlating adjusted mean scores for CG-CAHPS questions regarding patient behavior with ACGME Milestones professionalism domain ratings 6 months prior to graduation

eTable 5. Multivariable linear regression models correlating adjusted mean scores for CG-CAHPS questions regarding patient behavior with ACGME Milestones interpersonal and communication skills domain ratings 6 months prior to graduation

eTable 6. Multivariable linear regression models correlating Top Box scores for CG-CAHPS questions regarding patient behavior with ACGME Milestones professionalism domain ratings 6 months prior to graduation

eTable 7. Multivariable linear regression models correlating Top Box scores for CG-CAHPS questions regarding patient behavior with ACGME Milestones interpersonal and communication skills domain ratings 6 months prior to graduation

This supplemental material has been provided by the authors to give readers additional information about their work.

© 2025 Chen JX et al. *JAMA Network Open*.

eTable 1. Comparing the demographics of included and excluded physician cohorts who completed training at ACGME accredited programs between July 1, 2015, and June 30, 2019.

	Included physician cohort (Total N=1349)	Excluded physician cohort (Total N=49,798)	P value[A]
**Age range**			0.36
**21-25**	0 (0.0%)	52 (0.1%)	
**26-30**	753 (55.8%)	27,352 (54.9%)	
**31-35**	475 (35.2%)	17,376 (34.9%)	
**≥36**	121 (9.0%)	5,018 (10.1%)	
**Clinician gender**			**<0.001**
**Female**	804 (59.6%)	24,483 (49.2%)	
**Male**	522 (38.7%)	24,425 (49.0%)	
**Neither**	0 (0.0%)	34 (0.1%)	
**Prefer not to answer**	0 (0.0%)	19 (0.0%)	
**Missing**	23 (1.7%)	837 (1.7%)	
**Graduation year**			**0.001**
**2015**	14 (1.0%)	285 (0.6%)	
**2016**	324 (24.0%)	11,423 (22.9%)	
**2017**	344 (25.5%)	12,033 (24.2%)	
**2018**	369 (27.4%)	12,770 (25.6%)	
**2019**	298 (22.1%)	13,287 (26.7%)	
**Specialty**			**<0.001**
**Family medicine**	906 (67.2%)	13,716 (27.5%)	
**Internal medicine**	308 (22.8%)	30,973 (62.2%)	
**Obstetrics and gynecology**	135 (10.0%)	5,109 (10.3%)	
**Milestone rating**	**Average rating (SD)**	**Average rating (SD)**	**P-value[B]**
**Professionalism**	3.70 (0.56)	3.79 (0.51)	**<0.001**
**Interpersonal and communication skills**	3.77 (0.51)	3.83 (0.47)	**<0.001**

[A]*Chi-square tests for proportions*
[B]*Unpaired t-test*
SD=standard deviation

eTable 2. Multivariable linear regression models correlating **Top Box scores** for CG-CAHPS questions regarding physician behavior with ACGME Milestones professionalism domain ratings six months prior to graduation.

		CG-CAHPS Top Box score											
		Provider explained in way you understand		Provider showed respect for what you say		Provider spent enough time with you		Provider knew important info/ medical history		Provider listened carefully to you		Overall rating of provider (0 to 10)	
Risk factors		Adjusted mean change (95% CI)	p-value	Adjusted mean change (95% CI)	p-value	Adjusted mean change (95% CI)	p-value	Adjusted mean change (95% CI)	p-value	Adjusted mean change (95% CI)	p-value	Adjusted mean change (95% CI)	p-value
Milestone: professionalism domain rating	**≥3.5 vs. <3.5**	1.6 (0.8, 2.4)	**<.001**	1.5 (0.7, 2.2)	**<.001**	1.4 (0.6, 2.2)	**0.001**	2.9 (1.4, 4.5)	**<.001**	1.4 (0.6, 2.2)	**<.001**	2.9 (1.4, 4.3)	**<.001**
Sex	Male vs. Female	-1 (-1.8, -0.2)	**0.039**	-0.7 (-1.4, -0.1)	0.10	-0.3 (-1, 0.5)	0.80	-1.3 (-2.7, 0.2)	0.23	-0.7 (-1.5, 0)	0.17	-1.8 (-3.1, -0.4)	**0.038**
	Missing vs. Female	0.1 (-2, 2.3)		-0.1 (-2, 1.8)		-0.1 (-2.3, 2)		-0.7 (-4.7, 3.3)		-0.3 (-2.4, 1.8)		0.3 (-3.6, 4.1)	
Specialty	IM vs. FM	-1.4 (-2.3, -0.4)	**0.006**	-0.8 (-1.7, 0.1)	0.13	-0.5 (-1.5, 0.4)	0.33	-0.6 (-2.4, 1.3)	**0.04**	-0.9 (-1.8, 0.1)	0.10	-2.6 (-4.3, -0.9)	**<.001**
	OBGYN vs. FM	1.3 (-0.1, 2.6)		0.6 (-0.6, 1.7)		0.7 (-0.6, 2)		3.1 (0.7, 5.6)		0.8 (-0.5, 2.1)		2.9 (0.6, 5.2)	
Milestone: program mean of professionalism domain rating	Per one unit	-0.3 (-1.2, 0.7)	0.58	-0.2 (-1, 0.7)	0.69	0.1 (-0.9, 1)	0.85	-0.5 (-2.3, 1.3)	0.57	0 (-0.9, 0.9)	0.10	-0.6 (-2.3, 1.1)	0.50
CG-CAHPS: Clerks treat you with courtesy/respect	Per one unit	0.3 (0.2, 0.3)	**<.001**	0.2 (0.2, 0.3)	**<.001**	0.3 (0.2, 0.4)	**<.001**	0.5 (0.3, 0.6)	**<.001**	0.3 (0.2, 0.3)	**<.001**	0.4 (0.3, 0.5)	**<.001**
CG-CAHPS patient characteristics: % female	Per one unit	-1.7 (-4.5, 1.1)	0.24	-1.2 (-3.6, 1.3)	0.34	-1 (-3.8, 1.8)	0.49	-5.4 (-10.6, -0.2)	**0.040**	-2 (-4.7, 0.7)	0.15	-4.8 (-9.7, 0.1)	0.056
CG-CAHPS patient characteristics: % non-White	Per one unit	-1.2 (-3.1, 0.7)	0.20	-1.2 (-2.9, 0.4)	0.15	-3.9 (-5.8, -2)	**<.001**	3 (-0.5, 6.6)	0.090	-1.2 (-3.1, 0.6)	0.19	-1.4 (-4.7, 2)	0.42
CG-CAHPS patient characteristics: % non-English speaking at home	Per one unit	-10.4 (-17, -3.7)	**0.002**	-3.2 (-9, 2.7)	0.29	-11.3 (-18, -4.6)	**0.001**	-4.7 (-17.2, 7.8)	0.46	-5.7 (-12.2, 0.7)	0.083	-5.9 (-17.7, 5.8)	0.32

IM: internal medicine

FM: family medicine

OBGYN: obstetrics and gynecology

eTable 3. Multivariable linear regression models correlating **Top Box scores** for CG-CAHPS questions regarding physician behavior with ACGME Milestones interpersonal and communication skills domain ratings six months prior to graduation.

		CG-CAHPS Top Box score											
		Provider explained in way you understand		Provider showed respect for what you say		Provider spent enough time with you		Provider knew important info medical history		Provider listened carefully to you		Overall rating provider 0-10 by patients	
Risk factors		Adjusted mean change (95% CI)	p-value	Adjusted mean change (95% CI)	p-value	Adjusted mean change (95% CI)	p-value	Adjusted mean change (95% CI)	p-value	Adjusted mean change (95% CI)	p-value	Adjusted mean change (95% CI)	p-value
Milestone: interpersonal and communication skills domain rating	**≥3.5 vs. <3.5**	2.1 (1.2, 3)	**<.001**	1.8 (1, 2.6)	**<.001**	1.5 (0.6, 2.4)	**<.001**	3.2 (1.5, 4.9)	**<.001**	1.7 (0.8, 2.6)	**<.001**	3.5 (2, 5.1)	**<.001**
Sex	Male vs. Female	-1 (-1.8, -0.2)	**0.041**	-0.7 (-1.4, -0.1)	0.10	-0.3 (-1, 0.5)	0.80	-1.3 (-2.7, 0.2)	0.22	-0.7 (-1.5, 0)	0.17	-1.8 (-3.1, -0.4)	**0.039**
	Missing vs. Female	0 (-2.2, 2.2)		-0.2 (-2.1, 1.7)		-0.2 (-2.4, 2)		-0.8 (-4.8, 3.2)		-0.4 (-2.4, 1.7)		0.1 (-3.7, 3.9)	
Specialty	IM vs. FM	-1.6 (-2.6, -0.6)	**0.002**	-0.9 (-1.8, 0)	0.10	-0.4 (-1.5, 0.6)	0.38	-0.4 (-2.3, 1.6)	**0.037**	-0.8 (-1.8, 0.2)	0.14	-2.6 (-4.4, -0.7)	**0.001**
	OBGYN vs. FM	1.3 (0, 2.7)		0.6 (-0.5, 1.8)		0.8 (-0.6, 2.1)		3.2 (0.7, 5.7)		0.8 (-0.4, 2.1)		3 (0.7, 5.3)	
Milestone: program mean of ICS domain rating	Per one unit	-0.7 (-1.7, 0.3)	0.19	-0.4 (-1.2, 0.5)	0.41	0.1 (-0.9, 1.1)	0.84	-0.4 (-2.3, 1.4)	0.64	0 (-1, 1)	1.0	-0.7 (-2.4, 1.1)	0.46
CG-CAHPS: Clerks treat you with courtesy/respect	Per one unit	0.3 (0.2, 0.3)	**<.001**	0.2 (0.2, 0.3)	**<.001**	0.3 (0.2, 0.4)	**<.001**	0.5 (0.3, 0.6)	**<.001**	0.3 (0.2, 0.3)	**<.001**	0.4 (0.3, 0.5)	**<.001**
CG-CAHPS patient characteristics: % female	Per one unit	-1.7 (-4.5, 1.1)	0.23	-1.2 (-3.6, 1.3)	0.34	-1 (-3.8, 1.8)	0.50	-5.4 (-10.6, -0.2)	**0.041**	-2 (-4.7, 0.7)	0.15	-4.8 (-9.7, 0.1)	0.055
CG-CAHPS patient characteristics: % non-White	Per one unit	-1.3 (-3.1, 0.6)	0.19	-1.2 (-2.9, 0.4)	0.15	-3.9 (-5.8, -2)	**<.001**	3.1 (-0.4, 6.6)	0.087	-1.2 (-3, 0.6)	0.20	-1.4 (-4.7, 2)	0.42
CG-CAHPS patient characteristics: % non-English speaking at home	Per one unit	-10.3 (-16.9, -3.6)	**0.003**	-3 (-8.9, 2.8)	0.31	-11.1 (-17.8, -4.5)	**0.001**	-4.5 (-16.9, 8)	0.48	-5.6 (-12, 0.9)	0.092	-5.6 (-17.4, 6.1)	0.35

IM: internal medicine

FM: family medicine

OBGYN: obstetrics and gynecology

eTable 4. Multivariable linear regression models correlating **adjusted mean scores** for CG-CAHPS questions regarding physician behavior with ACGME Milestones professionalism domain ratings six months prior to graduation.

		CG-CAHPS adjusted mean score											
		Provider explained in way you understand		Provider showed respect for what you say		Provider spent enough time with you		Provider knew important info/ medical history		Provider listened carefully to you		Overall rating of provider (0 to 10)	
Risk factors		Adjusted mean change (95% CI)	p-value	Adjusted mean change (95% CI)	p-value	Adjusted mean change (95% CI)	p-value	Adjusted mean change (95% CI)	p-value	Adjusted mean change (95% CI)	p-value	Adjusted mean change (95% CI)	p-value
Milestone: professionalism domain rating	**≥3.5 vs. <3.5**	1 (0.5, 1.5)	**<.001**	0.8 (0.4, 1.3)	**<.001**	1 (0.4, 1.5)	**<.001**	2 (1, 3)	**<.001**	0.9 (0.3, 1.4)	**0.001**	1.2 (0.7, 1.8)	**<.0001**
Sex	Male vs. Female	-0.6 (-1.1, -0.1)	0.055	-0.5 (-0.9, 0)	0.099	-0.2 (-0.7, 0.3)	0.78	-0.9 (-1.8, 0.1)	0.19	-0.4 (-0.9, 0.1)	0.25	-0.6 (-1.1, 0)	0.12
	Missing vs. Female	-0.1 (-1.4, 1.2)		0 (-1.3, 1.2)		-0.1 (-1.5, 1.3)		-0.7 (-3.3, 1.9)		-0.2 (-1.6, 1.2)		-0.1 (-1.6, 1.5)	
Specialty	IM vs. FM	-0.7 (-1.3, -0.1)	**0.013**	-0.4 (-0.9, 0.2)	0.16	-0.3 (-1, 0.3)	0.28	-0.2 (-1.4, 1)	**0.019**	-0.4 (-1, 0.2)	0.15	-0.9 (-1.6, -0.2)	**0.003**
	OBGYN vs. FM	0.8 (0, 1.6)		0.6 (-0.2, 1.3)		0.6 (-0.3, 1.4)		2.3 (0.7, 3.9)		0.7 (-0.2, 1.5)		1.2 (0.2, 2.1)	
Milestone: program mean of professionalism domain rating	Per one unit	0 (-0.6, 0.5)	0.89	0 (-0.5, 0.5)	0.99	0 (-0.6, 0.6)	0.98	-0.1 (-1.3, 1)	0.81	0.1 (-0.5, 0.7)	0.73	-0.1 (-0.8, 0.5)	0.72
CG-CAHPS: Clerks treat you with courtesy/respect	Per one unit	0.3 (0.2, 0.4)	**<.001**	0.3 (0.2, 0.3)	**<.001**	0.3 (0.2, 0.4)	**<.001**	0.6 (0.4, 0.7)	**<.001**	0.3 (0.2, 0.4)	**<.001**	0.3 (0.2, 0.4)	**<.001**
CG-CAHPS patient characteristics: % female	Per one unit	-1.1 (-2.8, 0.6)	0.20	-1 (-2.6, 0.6)	0.23	-0.9 (-2.7, 1)	0.37	-4 (-7.4, -0.5)	**0.024**	-1.4 (-3.2, 0.4)	0.13	-1.1 (-3.1, 0.9)	0.27
CG-CAHPS patient characteristics: % non-White	Per one unit	-0.8 (-2, 0.4)	0.17	-0.8 (-1.9, 0.3)	0.15	-2.5 (-3.8, -1.3)	**<.001**	0.8 (-1.5, 3.1)	0.49	-1 (-2.2, 0.3)	0.12	-0.7 (-2, 0.6)	0.29
CG-CAHPS patient characteristics: % non-English speaking at home	Per one unit	-7 (-11, -2.9)	**<.001**	-2.4 (-6.2, 1.4)	0.22	-7.3 (-11.7, -2.8)	**0.001**	-5.4 (-13.6, 2.7)	0.19	-4.6 (-8.9, -0.3)	**0.035**	-2.6 (-7.3, 2.1)	0.28

IM: internal medicine

FM: family medicine

OBGYN: obstetrics and gynecology

eTable 5. Multivariable linear regression models correlating **adjusted mean scores** for CG-CAHPS questions regarding physician behavior with ACGME Milestones interpersonal and communication skills domain ratings six months prior to graduation.

		CG-CAHPS adjusted mean score											
		Provider explained in way you understand		Provider showed respect for what you say		Provider spent enough time with you		Provider knew important info medical history		Provider listened carefully to you		Overall rating provider 0-10 by patients	
Risk factors		Adjusted mean change (95% CI)	p-value	Adjusted mean change (95% CI)	p-value	Adjusted mean change (95% CI)	p-value	Adjusted mean change (95% CI)	p-value	Adjusted mean change (95% CI)	p-value	Adjusted mean change (95% CI)	p-value
Milestone: interpersonal and communication skills domain rating	**≥3.5 vs. <3.5**	1.2 (0.7, 1.8)	**<.001**	1 (0.5, 1.6)	**<.001**	1 (0.4, 1.6)	**<.001**	2.2 (1.1, 3.3)	**<.001**	1.1 (0.5, 1.6)	**<.001**	1.6 (0.9, 2.2)	**<.001**
Sex	Male vs. Female	-0.6 (-1.1, -0.1)	0.057	-0.5 (-0.9, 0)	0.10	-0.2 (-0.7, 0.3)	0.78	-0.9 (-1.8, 0.1)	0.19	-0.4 (-0.9, 0.1)	0.25	-0.6 (-1.1, 0)	0.12
	Missing vs. Female	-0.2 (-1.5, 1.2)		-0.1 (-1.3, 1.2)		-0.1 (-1.6, 1.3)		-0.8 (-3.4, 1.8)		-0.2 (-1.6, 1.1)		-0.1 (-1.7, 1.4)	
Specialty	IM vs. FM	-0.8 (-1.4, -0.1)	**0.007**	-0.4 (-1, 0.2)	0.14	-0.3 (-0.9, 0.4)	0.31	-0.1 (-1.4, 1.2)	**0.015**	-0.3 (-1, 0.3)	0.16	-1 (-1.7, -0.2)	**0.002**
	OBGYN vs. FM	0.9 (0.1, 1.7)		0.6 (-0.2, 1.3)		0.6 (-0.3, 1.5)		2.4 (0.8, 4)		0.7 (-0.1, 1.5)		1.2 (0.3, 2.1)	
Milestone: program mean of ICS domain rating	Per one unit	-0.2 (-0.8, 0.4)	0.49	-0.1 (-0.6, 0.5)	0.78	0 (-0.6, 0.7)	0.93	-0.2 (-1.4, 1)	0.77	0.1 (-0.5, 0.7)	0.75	-0.3 (-1, 0.4)	0.42
CG-CAHPS: Clerks treat you with courtesy/respect	Per one unit	0.3 (0.2, 0.4)	**<.001**	0.3 (0.2, 0.3)	**<.001**	0.3 (0.2, 0.4)	**<.001**	0.6 (0.4, 0.7)	**<.001**	0.3 (0.2, 0.4)	**<.001**	0.3 (0.2, 0.4)	**<.001**
CG-CAHPS patient characteristics: % female	Per one unit	-1.1 (-2.8, 0.6)	0.20	-1 (-2.6, 0.6)	0.23	-0.8 (-2.7, 1)	0.38	-3.9 (-7.4, -0.5)	**0.024**	-1.4 (-3.2, 0.4)	0.13	-1.1 (-3.1, 0.8)	0.27
CG-CAHPS patient characteristics: % non-White	Per one unit	-0.8 (-2, 0.3)	0.17	-0.8 (-1.9, 0.3)	0.16	-2.5 (-3.8, -1.3)	**<.001**	0.8 (-1.5, 3.2)	0.48	-0.9 (-2.2, 0.3)	0.13	-0.7 (-2, 0.6)	0.29
CG-CAHPS patient characteristics: % non-English speaking at home	Per one unit	-6.9 (-10.9, -2.8)	**<.001**	-2.3 (-6.1, 1.5)	0.23	-7.2 (-11.6, -2.8)	**0.002**	-5.3 (-13.5, 2.9)	0.21	-4.5 (-8.8, -0.2)	**0.039**	-2.5 (-7.1, 2.2)	0.30

IM: internal medicine

FM: family medicine

OBGYN: obstetrics and gynecology

eTable 6. Multivariable linear regression models correlating **Top Box scores** for CG-CAHPS questions regarding physician behavior with ACGME Milestones professionalism domain ratings six months prior to graduation.

		CG-CAHPS Top Box score											
		Provider explained in way you understand		Provider showed respect for what you say		Provider spent enough time with you		Provider knew important info/ medical history		Provider listened carefully to you		Overall rating of provider (0 to 10)	
Risk factors		Adjusted mean change (95% CI)	p-value	Adjusted mean change (95% CI)	p-value	Adjusted mean change (95% CI)	p-value	Adjusted mean change (95% CI)	p-value	Adjusted mean change (95% CI)	p-value	Adjusted mean change (95% CI)	p-value
Milestone: professionalism domain rating	**≥4 vs. <4**	1.6 (0.9, 2.2)	**<.001**	1.2 (0.6, 1.8)	**<.001**	1.1 (0.5, 1.8)	**<.001**	1.9 (0.7, 3.1)	**0.002**	1.4 (0.8, 2)	**<.001**	2.4 (1.3, 3.5)	**<.001**
Sex	Male vs. Female	-1 (-1.8, -0.2)	**0.041**	-0.7 (-1.4, -0.1)	0.098	-0.3 (-1, 0.5)	0.80	-1.3 (-2.7, 0.2)	0.23	-0.7 (-1.5, 0)	0.18	-1.8 (-3.1, -0.4)	**0.038**
	Missing vs. Female	0.2 (-1.9, 2.4)		0 (-1.9, 1.9)		-0.1 (-2.2, 2.1)		-0.5 (-4.5, 3.5)		-0.2 (-2.3, 1.9)		0.4 (-3.4, 4.2)	
Specialty	IM vs. FM	-1.7 (-2.7, -0.7)	**0.001**	-0.9 (-1.8, -0.1)	0.079	-0.7 (-1.7, 0.3)	0.28	-0.5 (-2.4, 1.4)	**0.049**	-1.2 (-2.1, -0.2)	0.038	-2.9 (-4.7, -1.1)	**<.001**
	OBGYN vs. FM	1.2 (-0.1, 2.5)		0.5 (-0.7, 1.7)		0.7 (-0.7, 2)		3 (0.6, 5.5)		0.7 (-0.6, 2)		2.8 (0.5, 5.1)	
Milestone: program mean of professionalism domain rating	Per one unit	-0.6 (-1.5, 0.4)	0.26	-0.3 (-1.1, 0.6)	0.50	0 (-1, 1)	0.98	-0.4 (-2.2, 1.4)	0.64	-0.3 (-1.2, 0.7)	0.59	-0.8 (-2.5, 0.9)	0.34
CG-CAHPS: Clerks treat you with courtesy/respect	Per one unit	0.3 (0.2, 0.3)	**<.001**	0.2 (0.2, 0.3)	**<.001**	0.3 (0.2, 0.4)	**<.001**	0.5 (0.3, 0.6)	**<.001**	0.3 (0.2, 0.3)	**<.001**	0.4 (0.3, 0.5)	**<.001**
CG-CAHPS patient characteristics: % female	Per one unit	-1.8 (-4.6, 1)	0.20	-1.3 (-3.8, 1.1)	0.30	-1.1 (-3.9, 1.7)	0.44	-5.6 (-10.8, -0.4)	0.036	-2.1 (-4.8, 0.6)	0.12	-5 (-10, -0.1)	**0.045**
CG-CAHPS patient characteristics: % non-White	Per one unit	-1.1 (-3, 0.7)	0.23	-1.1 (-2.8, 0.5)	0.18	-3.8 (-5.7, -1.9)	**<.001**	3.2 (-0.3, 6.7)	0.073	-1.1 (-3, 0.7)	0.22	-1.2 (-4.5, 2.1)	0.47
CG-CAHPS patient characteristics: % non-English speaking at home	Per one unit	-11.1 (-17.8, -4.5)	**0.001**	-3.8 (-9.6, 2.1)	0.20	-11.9 (-18.6, -5.2)	**<.001**	-5.9 (-18.4, 6.6)	0.35	-6.4 (-12.9, 0)	0.051	-7.2 (-18.9, 4.6)	0.23

IM: internal medicine

FM: family medicine

OBGYN: obstetrics and gynecology

eTable 7. Multivariable linear regression models correlating **Top Box scores** for CG-CAHPS questions regarding physician behavior with ACGME Milestones interpersonal and communication skills domain ratings six months prior to graduation.

		CG-CAHPS Top Box score											
		Provider explained in way you understand		Provider showed respect for what you say		Provider spent enough time with you		Provider knew important info medical history		Provider listened carefully to you		Overall rating provider 0-10 by patients	
Risk factors		Adjusted mean change (95% CI)	p-value	Adjusted mean change (95% CI)	p-value	Adjusted mean change (95% CI)	p-value	Adjusted mean change (95% CI)	p-value	Adjusted mean change (95% CI)	p-value	Adjusted mean change (95% CI)	p-value
Milestone: interpersonal and communication skills domain rating	**≥4 vs. <4**	1.5 (0.8, 2.1)	**<.001**	1.0 (0.4, 1.6)	**<.001**	0.9 (0.3, 1.6)	**0.006**	2.2 (1, 3.4)	**<.001**	1.1 (0.5, 1.7)	**<.001**	2.1 (1, 3.3)	**<.001**
Sex	Male vs. Female	-1 (-1.8, -0.3)	**0.028**	-0.8 (-1.5, -0.1)	0.078	-0.3 (-1.1, 0.5)	0.76	-1.3 (-2.8, 0.1)	0.19	-0.8 (-1.5, 0)	0.15	-1.8 (-3.2, -0.5)	**0.029**
	Missing vs. Female	0.2 (-2, 2.3)		0 (-1.9, 1.9)		-0.1 (-2.2, 2.1)		-0.6 (-4.6, 3.4)		-0.2 (-2.3, 1.9)		0.3 (-3.5, 4.2)	
Specialty	IM vs. FM	-1.9 (-3, -0.8)	**<.001**	-0.9 (-1.9, 0)	0.10	-0.5 (-1.6, 0.6)	0.34	-0.8 (-2.8, 1.3)	0.031	-0.9 (-2, 0.1)	0.10	-2.8 (-4.7, -0.8)	**0.001**
	OBGYN vs. FM	1.4 (0, 2.7)		0.6 (-0.5, 1.8)		0.8 (-0.6, 2.1)		3.2 (0.7, 5.7)		0.8 (-0.4, 2.1)		3 (0.7, 5.3)	
Milestone: program mean of ICS domain rating	Per one unit	-0.8 (-1.8, 0.2)	0.13	-0.3 (-1.2, 0.6)	0.52	0.1 (-0.9, 1.1)	0.85	-0.6 (-2.5, 1.3)	0.52	0 (-1, 1)	0.95	-0.6 (-2.4, 1.2)	0.50
CG-CAHPS: Clerks treat you with courtesy/respect	Per one unit	0.3 (0.2, 0.3)	**<.001**	0.2 (0.2, 0.3)	**<.001**	0.3 (0.2, 0.4)	**<.001**	0.5 (0.3, 0.6)	**<.001**	0.3 (0.2, 0.3)	**<.001**	0.4 (0.3, 0.5)	**<.001**
CG-CAHPS patient characteristics: % female	Per one unit	-2 (-4.8, 0.8)	0.16	-1.4 (-3.8, 1.1)	0.27	-1.1 (-4, 1.7)	0.42	-5.9 (-11.1, -0.7)	**0.028**	-2.2 (-4.9, 0.5)	0.11	-5.2 (-10.2, -0.3)	**0.038**
CG-CAHPS patient characteristics: % non-White	Per one unit	-1.1 (-3, 0.8)	0.25	-1.1 (-2.8, 0.6)	0.19	-3.8 (-5.7, -1.9)	**<.001**	3.3 (-0.2, 6.8)	0.065	-1.1 (-2.9, 0.7)	0.25	-1.1 (-4.4, 2.2)	0.52
CG-CAHPS patient characteristics: % non-English speaking at home	Per one unit	-10.9 (-17.5, -4.2)	**0.001**	-3.5 (-9.4, 2.3)	0.24	-11.6 (-18.3, -4.9)	**<.001**	-5.6 (-18.1, 6.9)	0.38	-6.1 (-12.5, 0.4)	0.066	-6.7 (-18.4, 5.1)	0.27

IM: internal medicine

FM: family medicine

OBGYN: obstetrics and gynecology
