# Supplementary material for: Trainee-Physician Milestones Ratings and Patient Experience Surveys in Early Unsupervised Practice
Source: JAMA Netw Open. 2025 Oct 8;8(10):e2536380. doi: 10.1001/jamanetworkopen.2025.36380 (PMC12509016; doi:10.1001/jamanetworkopen.2025.36380)
Supplement: Supplement 2. — Data Sharing Statement [file jamanetwopen-e2536380-s002.pdf]

# Data Sharing Statement

Chen. Trainee-Physician Milestones Ratings and Patient Experience Surveys in Early Unsupervised Practice. *JAMA Netw Open*. Published October 08, 2025. doi:10.1001/jamanetworkopen.2025.36380

**Data**
**Data available:** No

**Additional Information**
**Explanation for why data not available:** The study was performed by the Data Use Agreement between ACGME and Press Ganey.
